# Green-synthesized ZnO nano-crystallites as an electrochemical sensor for efficient detection of heavy metal

**DOI:** 10.1039/d6ra01784c

**Published:** 2026-07-02

**Authors:** Sumaya Tabassum, Md. Abu Saeed, Promit Debnath, Nilufar Yasmin Liza, Sanjida Khan, Muhammad Shahriar Bashar, Suravi Islam, Samina Ahmed, Md. Sahadat Hossain

**Affiliations:** a Institute of Glass & Ceramic Research and Testing, Bangladesh Council of Scientific and Industrial Research (BCSIR) Dhaka-1205 Bangladesh saz8455@gmail.com shanta_samina@yahoo.com; b Department of Natural Sciences, BGMEA University of Fashion and Technology Nishatnagar, Turag Dhaka-1230 Bangladesh; c Department of Materials & Metallurgical Engineering, Bangladesh University of Engineering & Technology Dhaka-1000 Bangladesh; d University of Chinese Academy of Sciences Beijing-100049 China; e Institute of Leather Engineering and Technology, University of Dhaka Dhaka-1000 Bangladesh; f Institute of Fuel Research and Development, Bangladesh Council of Scientific and Industrial Research (BCSIR) Dhaka 1205 Bangladesh

## Abstract

Green-synthesized nanomaterials have garnered increased interest in mitigating the issue of freshwater scarcity worldwide. So, focusing on this issue, bay leaf extract (*Laurus nobilis*) was employed to synthesize ZnO NPs, which were able to detect the heavy metal (Cd^2+^) in aqueous solution. The synthesized material was characterized by XRD, FTIR spectroscopy, FE-SEM equipped with EDX, and TGA. The structure of ZnO (hexagonal wurtzite) was assured from the X-ray diffractogram, and the determined crystallite size was within 4–127 nm depending on several model equations such as the Sahadat–Scherrer Method, Monshi–Scherrer Method, Linear Straight-line Method of Scherrer's equation, Halder–Wagner method, Williamson–Hall plots, and size-strain plot. TGA revealed the weight loss (three stages), whereas the SEM micrograph confirmed the presence of small spherical particles. The EDX assured the presence of Zn (84.45 wt%) and O (15.55 wt%). A glassy carbon working electrode fabricated with ZnO NPs was able to detect heavy metal (Cd) through the DPASV electrochemical technique with a linear range of 0.1–7 ppm.

## Abstract 2

*Laurus nobilis* extract-mediated ZnO nanoparticles were used to modify a glassy carbon electrode for the electrochemical detection of heavy metal (Cd^2+^) using differential pulse anodic stripping voltammetry (DPASV).

## Graphical abstract

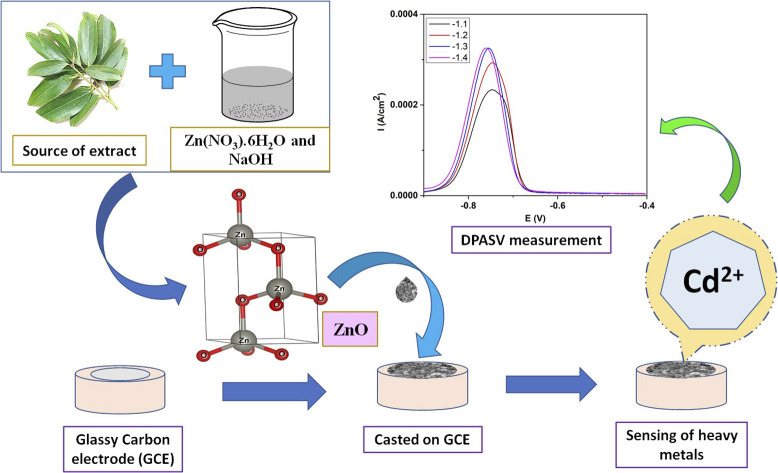

## 1. Introduction

The rapid rise in population, with the global population projected to be 9.8 billion by 2050, and industrialization, have intensified environmental pollution, a major issue.^1^ Access to clean water has become increasingly scarce, affecting approximately two billion people, or 26% of the world's population, who face a lack of regulated drinking water due to chemical pollutants, microbial pollution, and inadequate infrastructure. Contamination of water by heavy metals (Cd, Pb, Hg, Cu) causes adverse health problems and an imbalanced ecosystem.^2,3^ Cadmium ions are non-biodegradable and can slowly accumulate inside the human body. The Cd in contact with the soil also decreases the fertility of the soil, and in this way, Cd can also enter the food chain. As a result, the World Health Organization has declared Cd as a carcinogen that can cause cancer, alter DNA, and damage the cell membrane; besides, its half-life is more than 10 years.^4,5^

Research on nanomaterials has been spreading swiftly in different fields (biosensors, biomedical, solar cells, drug delivery, catalysis, textiles, food, water purification, cosmetics, and medicine.^6^ The most common metals or metal oxides are copper, silver, iron, nickel, titanium dioxide, iron oxide, and zinc oxide.^7^ Different methods (chemical, biological, and physical methods) for the synthesis of metal oxides are well known, but all these methods require high energy, are expensive, and not environmentally friendly.^8^ To overcome these obstacles, green synthesis is the most favorable approach because it is safe, cost-effective, and non-hazardous to the ecosystem.^9^ Among all nanoparticles, ZnO NPs have captured greater attention because of their easy synthesis, safety to produce, and low cost. Moreover, the US Food and Drug Administration has declared ZnO NPs as one of the most risk-free metal oxides.^10,11^ Green-synthesized ZnO nanoparticles apply to diverse fields, including chemical sensors, solar cells, gas sensors, biosensors, photodetectors, wound healing, and anti-inflammation.^12,13^

Several parts of plants, such as the seed, leaf, root, bark, stem, flower, fruit, and the extract of peel, are used to synthesize the ZnO NPs. The plant extract-synthesized NPs give more stability to the nanoparticles. Plant extracts contain phytochemicals such as terpenoids, polyphenols, sugars, and flavonoid alkaloids that enhance their reducing and capping properties. ZnO NPs have been synthesized from the Lauraceae family bay leaf (*Laurus nobilis*), which is a popular spice in all Asian and Western countries as a culinary spice.^14^ Bay leaf contains volatile oils (linalool, 1,8-cineole, α-terpinyl acetate), phenolic compounds (flavonol, procyanidin trimmer, flavone, epicatechin), and volatile active compounds (neral, α-pinene, myrcene, methyl chavicol, β-pinene).^15^ Because of the presence of these components in bay leaf, it has anti-oxidant, antiseptic, anti-cancer, and digestive properties, so it can be used as a herbal medicine for the treatment of indigestion, perspiration, rheumatism, diabetes, and migraine. So, green-synthesized NPs are biodegradable, non-toxic, and more reactive due to a high surface-to-volume ratio, great catalytic activity, and semiconducting properties; as a result, they have attracted greater attention in heavy metal detection.^16^

Conventional methods, such as AAS, UV-vis spectroscopy, mass spectroscopy, ICP-OES, and ICP-MS, are not only costly but also require regular maintenance.^17–19^ Overcoming all these obstacles, the electrochemical sensing technique, such as differential pulse anodic stripping voltammetry (DPASV), square wave voltammetry (SWV), cyclic voltammetry (CV), electrochemical impedance spectroscopy, and, linear sweep anodic stripping voltammetry (LSASV), has become applicable for heavy-metal detection (Cd, Pd, Hg) exhibiting more accuracy and within very short times.^20,21^ As an electrochemical sensor, green-synthesized ZnO NPs-modified sensors are effective due to their uniqueness, including nano-sized particles with a high surface area, which facilitates the detection of heavy metals in aqueous media. Most exploration has focused on the utilization of green-synthesized ZnO NPs for antimicrobial activity and photocatalytic applications as a catalyst. But the electrochemical sensor modified by bay leaf extracted ZnO NPs for cadmium detection through the electrochemical technique (DPASV-Differential pulse anodic stripping voltammetry) has remained unexplored in earlier studies.

The synthesis of ZnO NPs utilizing bay leaf extract (*Laurus nobilis*) has been the main focus of this exploration, with estimation of crystallite size using several model equations, which can also detect cadmium in aqueous medium through electrochemical methods. This exploration involves the development and testing of a green sensor, an environmentally friendly device designed to accurately detect heavy metals.

## 2. Materials and methodology

### 2.1. Materials

The source of fresh bay leaves (*Laurus nobilis*) was inside the garden of BCSIR's official area. To continue the whole reaction process, deionized water was used. Zinc-nitrate hexahydrate (Zn(NO_3_)_2_·6H_2_O, 96% purity) was from LOBA CHEMIE PVT. LTD. Sodium hydroxide (99%) pellets were purchased from a local supplier, and the company name was Sigma-Aldrich (Germany).

### 2.2. Synthesis methods

#### 2.2.1. Bay leaf extract preparation

Fresh leaves of *Laurus nobilis* were thoroughly rinsed with deionized water to eliminate surface contaminants, sun-dried, and subsequently ground into a fine powder using a mortar and pestle. Approximately 5 g of the fine powder was placed into a 250 mL beaker, mixed with 50 mL of deionized water, and subsequently heated at 80 °C for 20 minutes. The aqueous extract was obtained by filtering the mixture with Whatman No. 1 filter paper. The extract was cooled to room temperature, kept at 4 °C, and subsequently used for the synthesis of ZnO NPs.

#### 2.2.2. Preparation of ZnO NPs

**Fig. 1:**
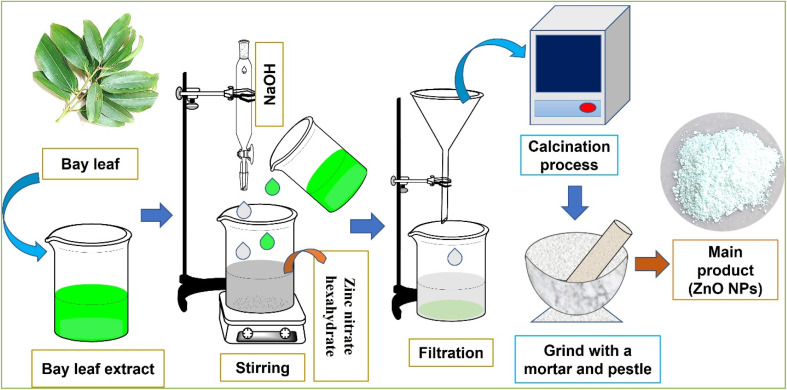
Synthesis of ZnO NPs utilizing bay leaf extract.

0.05 M aqueous Zn(NO_3_)_2_·6H_2_O solution with the required amount of *Laurus nobilis* leaf extract in a 250 mL beaker was taken for the preparation of the desired product. For carrying out the reaction, 0.2 M NaOH solution was added dropwise until the pH was adjusted to 12. A magnetic stirrer was used to stir the mixture for 3 hours at ambient temperature, followed by filtration. The resulting light-orange precipitate was dried overnight at 60 °C in a laboratory oven. The dried product was calcined at 500 °C for 3 hours, yielding a white ZnO NP powder. The final product was collected, cooled, and kept in a desiccator for further use. Fig. 1 depicts the synthesis process of the final product.

### 2.3. Instrumental characterization

The crystal structure parameters were determined using a Rigaku SE XRD diffractometer with a radiation source of Cu kα (*λ* = 1.5419 Å). Data acquisition was carried out at 20 °C in the range of 2*θ* (20–70°), with a step increment of 0.01°, operating at 50 kV voltage with 40 mA current. Functional groups associated with surface biomolecules were analyzed by FTIR spectra (IR-Prestige 21 spectrometer, Shimadzu, Japan), recorded over 400–4000 cm^−1^ (30 scans, 4 cm^−1^ resolution). The thermal behavior and stability were performed by TGA (8000, PerkinElmer) under a nitrogen atmosphere within a temperature range of 50–800 °C. Morphology and elemental composition were examined by SEM (Carl Zeiss, US) operating at 25 kV, equipped with EDX (AMETEK, US; operating at 15 kV). For electrochemical analysis, the three-electrode system was used (Ag/AgCl as the reference electrode, glassy carbon as the working electrode, Pt-wire as the counter electrode), and the model of the instrument was Corrtest CS300 (China). A 0.1 M NaCl solution was used as an electrolyte for the electrochemical experiment.

## 3. Results and discussion

### 3.1. XRD analysis

**Fig. 2:**
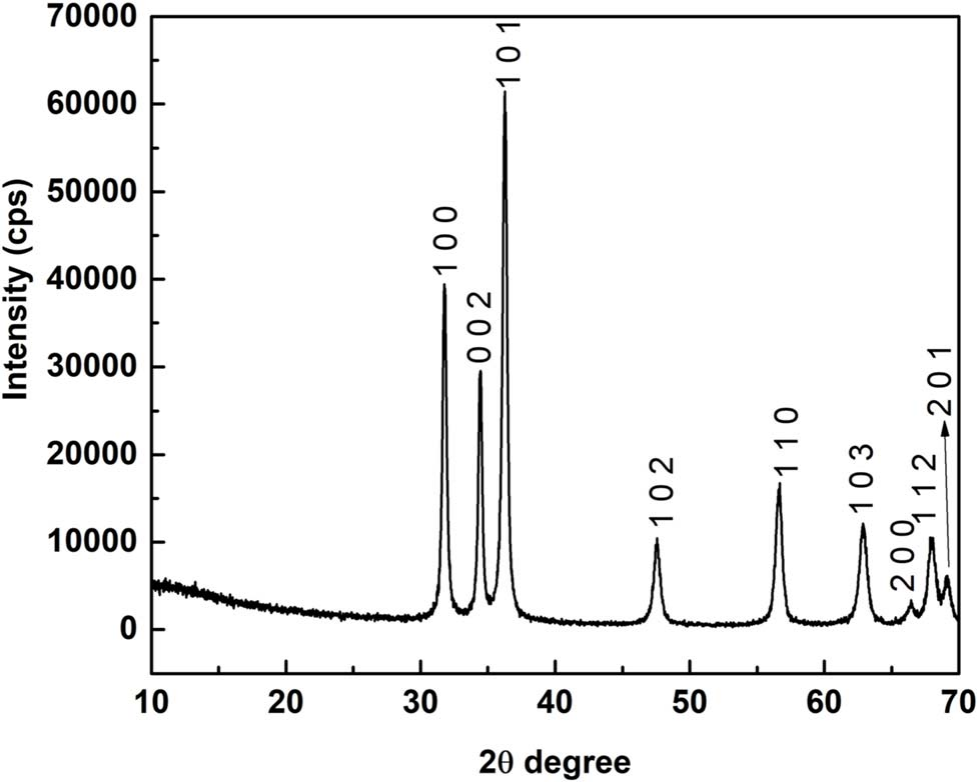
X-ray diffractogram of green-synthesized ZnO NPs.

Crystalline morphology and anisotropy are present in the produced ZnO nanoparticles. Fig. 2 demonstrates the intense and sharp XRD pattern for ZnO nanoparticles generated *via* the green approach, which represents the 2*θ* values at the 31.770, 34.420, 36.251, 47.540, 56.600, 62.860, 66.380, 67.950, and 69.090 which were assigned to the planes such as (1 0 0), (0 0 2), (1 0 1), (1 0 2), (1 1 0), (1 0 3), (2 0 0), (1 1 2), and (2 0 1) which are in good agreement with the standard database (ICDD card no. #00-036-1451) and are indexed as a typical wurtzite type crystal structure of ZnO. The (101) plane exhibited the highest relative intensity in the XRD pattern, indicating anisotropic growth and preferred orientation of the crystallites due to agglomeration, as epitaxial growth along the *y*-axis in the (0 0 1) direction is characteristic of wurtzite-structured materials. ICDD confirmed that it was ZnO NPs. The narrow peal width, which results from ZnO NP's high-intensity diffraction pattern, demonstrates the precise hexagonal wurtzite makeup of ZnO.

For the ZnO nanoparticles, the Debye–Scherrer's equation average crystallite size was used to calculate for the (1 0 1) plane, stated as eqn (1).^22^1
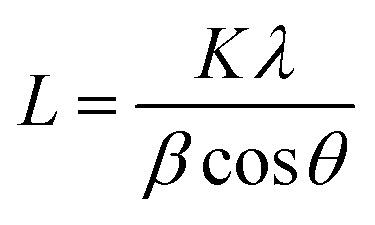
where *λ* indicates a wavelength of X-ray radiation, *L* is for the crystallite size, and the full-width half-maximum is *β*, and the diffraction angle is *θ*. No extra peak was obtained, which ensures the purity of the synthesized material. The crystallite size calculated based on Debye–Scherrer's equation was 21.5996 nm. Table 1 exposes the ZnO NPs' standard valued diffraction angles and planes with green-synthesized ZnO NPs. The latter's crystallographic parameters were computed based on eqn (2)–(5):^23^2
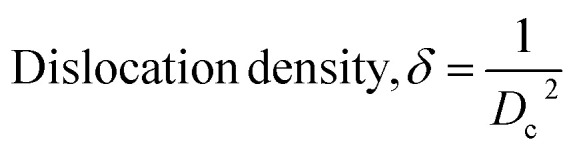
3
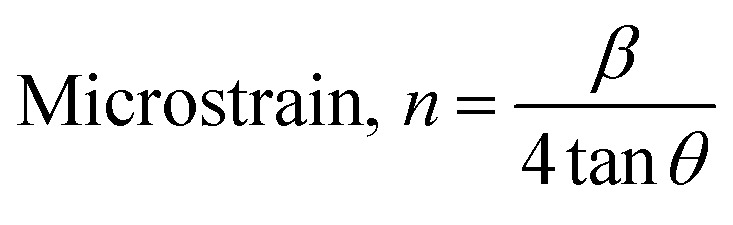
4
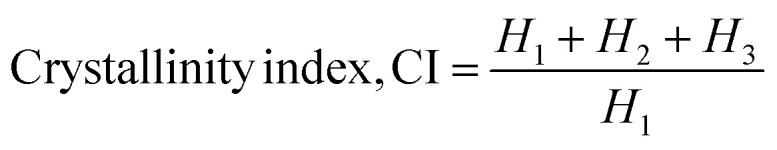


**Table 1:** ZnO NPs' standard valued diffraction angles and planes with synthesized ZnO NPs

Diffraction angle (2*θ*)		Planes (*h k l*)
ZnO NPs	Standard value, card no:#00-036-1451	
31.740	31.770	1 0 0
34.434	34.420	0 0 2
36.251	36.251	1 0 1
47.521	47.540	1 0 2
56.576	56.600	1 1 0
62.795	62.860	1 0 3
66.380	66.380	2 0 0
67.913	67.950	1 1 2
69.034	69.090	2 0 1

Lattice parameter equation (monoclinic)5
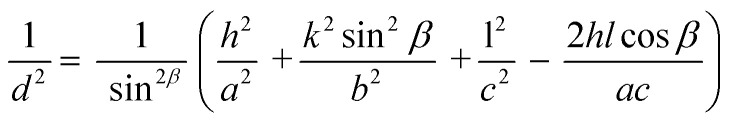


In eqn (2)–(5), *b* is the Full width at Half maximum in radians, *θ* is the angle of diffraction, *H*_1_, *H*_2_, and *H*_3_ are the heights of the three strongest peaks, *K* is Scherrer's constant, and *a*, *b*, *c*, *h*, *k*, and *l* are the lattice parameters of the crystals. The evaluated data, based on eqn (2)–(5), are listed in Table 2.

**Table 2:** Estimated crystallographic parameters

Crystallographic parameters	Deduced value
Crystallite size, *L* (nm)	21.5996
Dislocation density (10^15^ lines per m^2^)	0.0021
Lattice parameters (Å)	*a* = *b* = 3.2526, *c* = 5.2050
Microstrain	0.2955
Crystallinity index	2.0977

Three planes (1 0 0), (0 0 2), and (1 1 0) were compared to a specific plane (1 0 1) to calculate the preference growth (eqn (7)), and in that case, the relative intensity (eqn (6)) was taken into account, in which the value of RI_st_ (relative intensities of the standard) and RI_s_ (relative intensities of the synthesized samples) were used.^24^ Based on the same method and equations, the preference growth and relative intensity of the (0 0 2), (1 0 0), and (1 1 0) planes were calculated, and the computed data are shown in Table 3. The thermodynamically advantageous plane was symbolized by the positive value of preference growth, while the negative value implied the thermodynamically unfavorable plane. Significantly, there was no presence of extra peaks, so the end product is associated exclusively with ZnO nanostructures.6
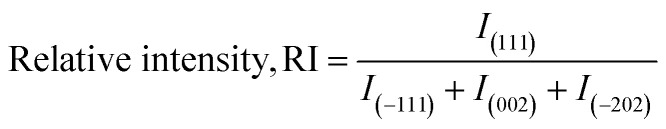
7
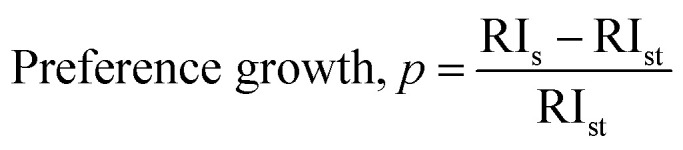


**Table 3:** Estimated preference growth and relative intensity

Plane	The other three planes	Relative intensity		Preference growth
		Standard, RI_st_	Sample, RI_s_	
1 0 1	0 0 2, 1 0 0, 1 1 0	0.7518	0.7609	0.0120
0 0 2	1 0 1, 1 0 0, 1 1 0	0.2328	0.1906	−0.1809
1 0 0	1 0 1, 0 0 2, 1 1 0	0.3238	0.3350	0.0344
1 1 0	1 0 1, 0 0 2, 10 0	0.1592	0.1859	0.1678

### 3.2. Estimation of crystallite size using several model equations

**Fig. 3:**
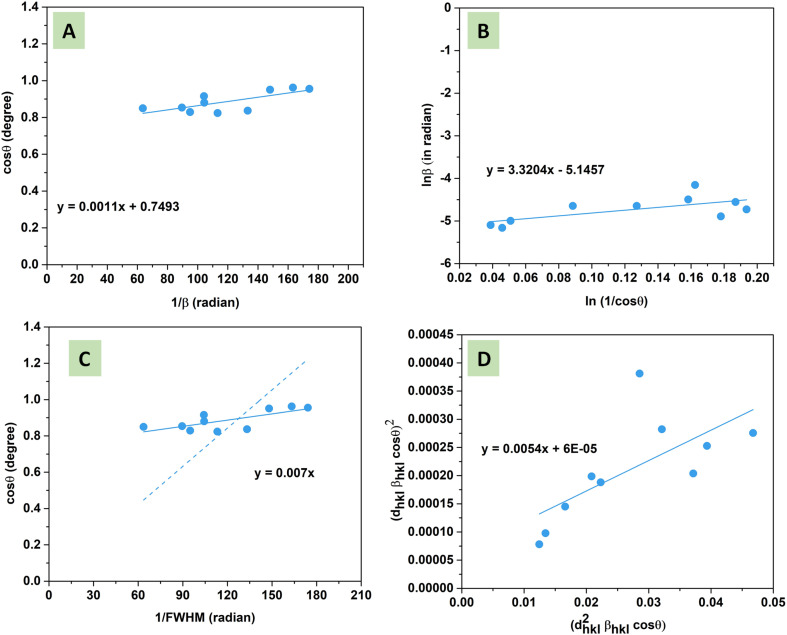
Linear straight-line method of Scherrer equation (A), Monshi–Scherrer method (B), Sahadat–Scherrer model (C), size-strain plot method (D).

**Fig. 4:**
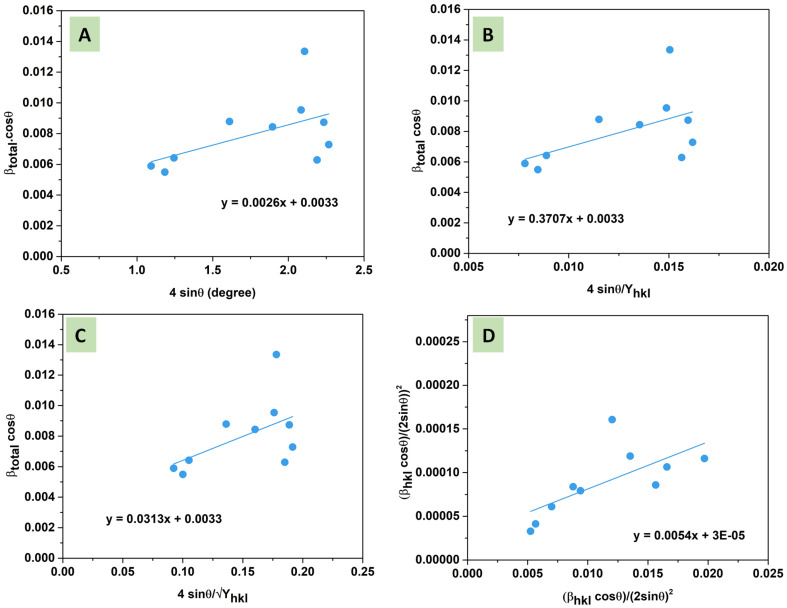
UDM (A), USDM (B), UDEDM (C), Halder–Wagner method (D).

The six-model with three sub-model equations (Table 4) was used, and each equation was correlated with the fundamental straight-line equation (*y* = *mx* + *c*) for computing the crystallite size, stress, strain, and energy density. In Table 4, eqn (8)–(13), *θ* = diffraction angle, *K* = Scherrer's constant, *Y*_hkl_ = modulus of elasticity, which is called the Young modulus, *µ* = energy density, FWHM (*β*_total_) = Full width at Half Maximum, *D* = crystallite size, *ε* = strain, *σ* = stress, and *λ* = wavelength of the X-ray source. A detailed description of each model can be found in the existing literature.^25,26^Fig. 3 and 4 represent the model based on equations from which the computed crystallite size is included in Table 5.

**Table 4:** Several model equations for the estimation of crystallite size

Equation no.	Name of the model	Equation	Fig. no.
8	Linear straight-line method of Scherrer's equation	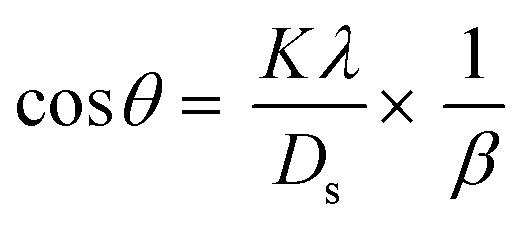	3A
9	Monshi–Scherrer method	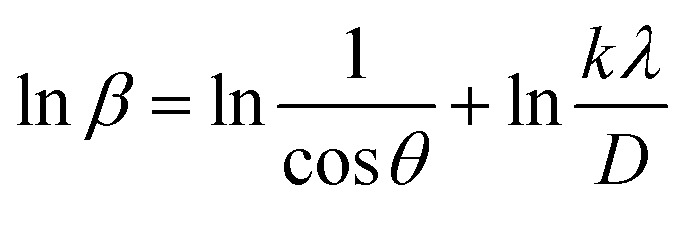	3B
10	Sahadat–Scherrer method	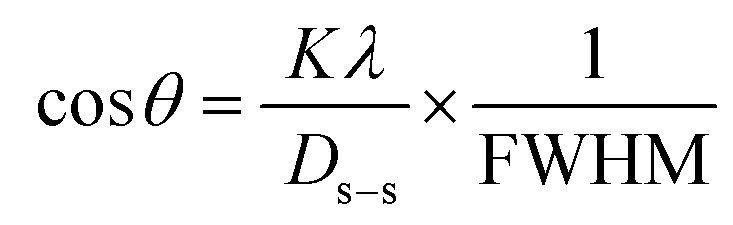	3C
11	Size-strain plot method	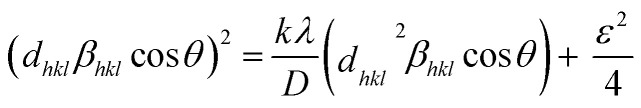	3D
12	Williamson–Hall plot	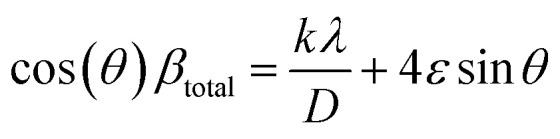	4A
		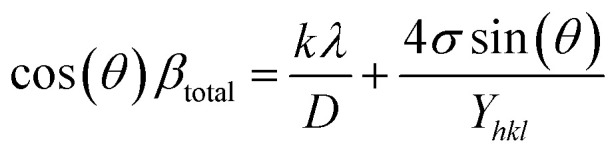	4B
		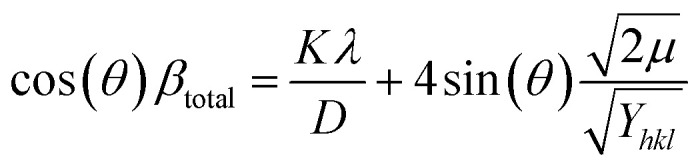	4C
13	Halder–Wagner method	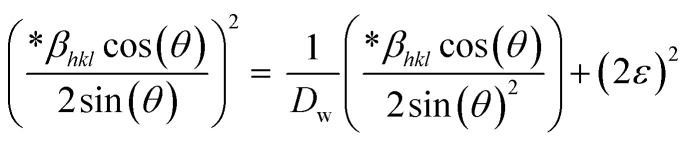	4D

**Table 5:** Estimated crystallite size depending on several model equations

Model name		Crystallite size (nm), strain (*ε*) (N m^−2^), stress (*σ*) (N m^−2^), energy density (*µ*) (kJ m^−3^)
LSMS		126.0490
MSM		23.1057
S–S		19.8077
SSP		25.6766, *ε* = 0.0154
W–H	UDM	42.0163, *ε* = 0.0026
	USDM	42.0163, *ε* = 0.0023, *σ* = 3.70 × 10^8^
	UDEDM	4.4298, *ε* = 1.148 × 10^9^, *µ* = 489.845
HWM		18.5185

### 3.3. FTIR analysis

**Fig. 5:**
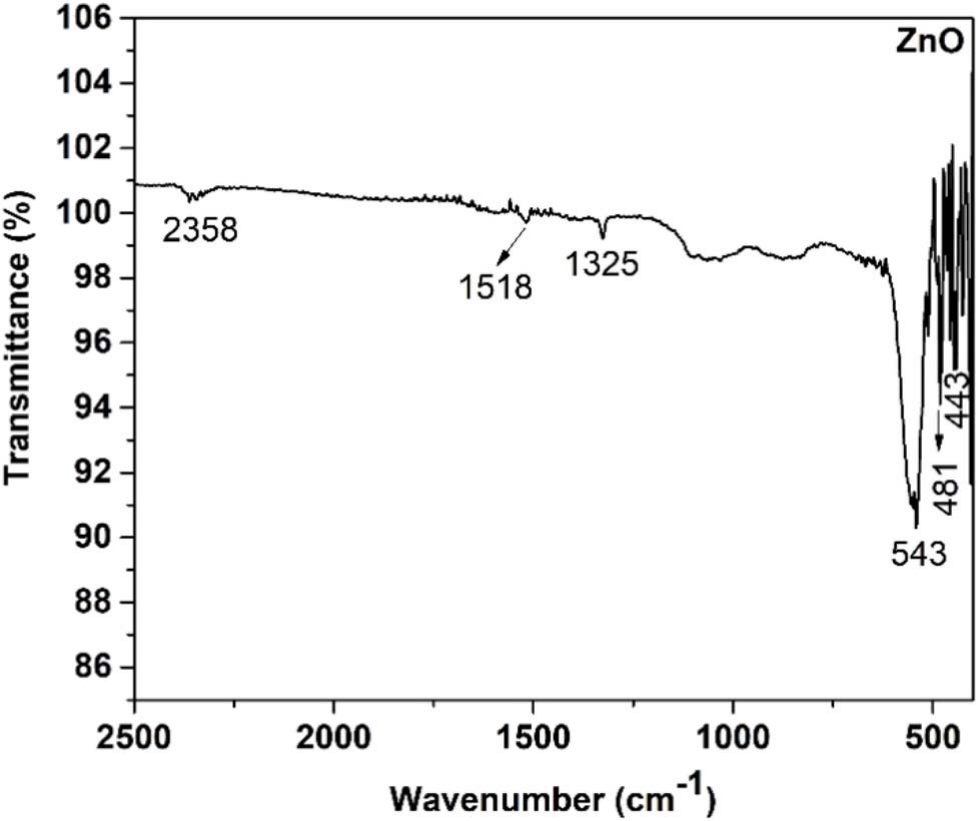
FTIR spectrum of the green-synthesized ZnO NPs.

The functional groups in the synthesized material (visualized in Fig. 5) were identified using FTIR spectroscopy. The peaks at 2358, 1518, and 543 cm^−1^ are responsible for the vibrational modes of CO_2_, the bending vibration of H–O–H, and Zn–O stretching vibration, respectively.^27^ The presence of bonding between metal and oxygen atoms is indicated by the bands that are 400 and 750 cm^−1^. Zn–O stretching of ZnO in the 543–443 cm^−1^ region was identified as the cause of the major absorption band's FTIR spectra. The band's appearance at 481 cm^−1^ verified the synthesis of ZnO NPs and confirmed a stretching frequency of Zn–O bonds.^28^

### 3.4. Thermogravimetric analysis

**Fig. 6:**
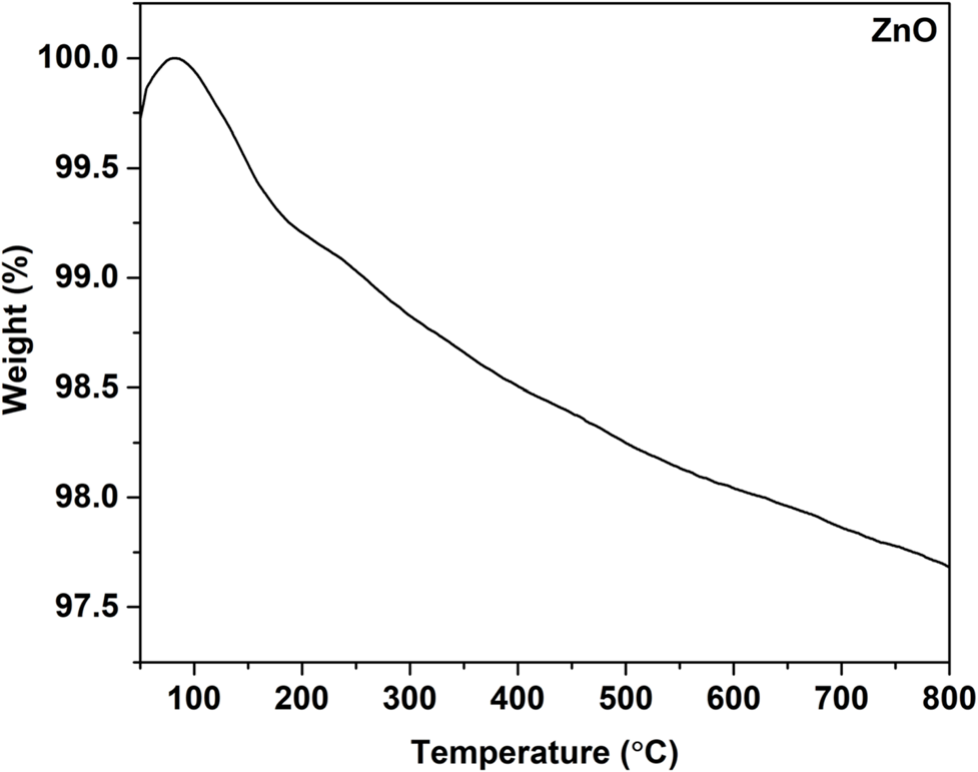
Thermogravimetric analysis of ZnO NPs.

Fig. 6 shows the thermogravimetric analyzer used to determine the weight loss (three stages) of ZnO NPs. The higher thermal stability may be attributed to the high crystallinity of the material. The small loss indicates fewer defects in the materials. The weight loss around the temperature range (90 °C–120 °C) was ∼17.59%, which is attributed to the loss of chemisorbed –OH groups by evaporation. In continuation, the weight loss around the temperature range (240 °C–260 °C) was ∼39.28%, which is attributed to the loss of acetate groups, and ensured the formation of ZnO structure. This was first introduced by the biomolecules on the surface of the produced ZnO NPs, which burned or broke down.

### 3.5. Surface morphology analysis

**Fig. 7:**
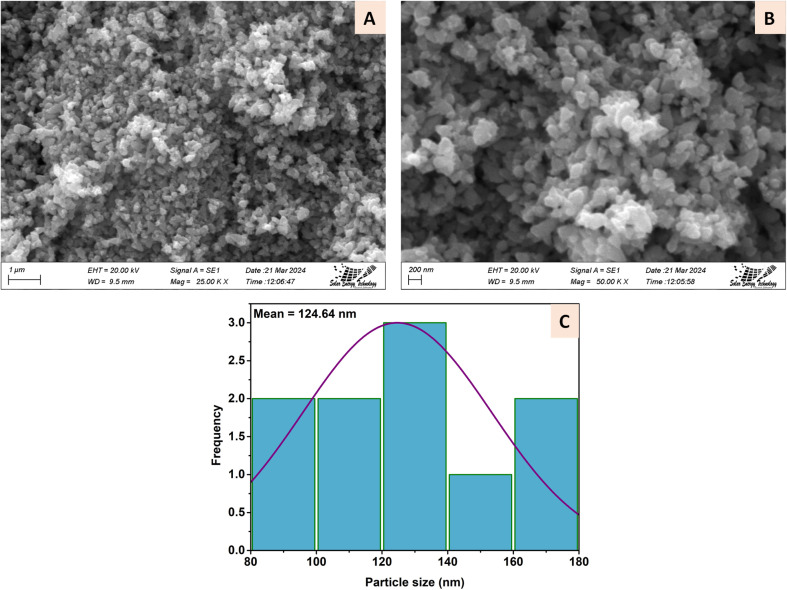
SEM images (A and B) of the green-synthesized ZnO NPs and histogram analysis (C).

The SEM micrograph of ZnO nanocrystalline particles is shown in Fig. 7(A and B) at different magnifications. These pictures indicated individual ZnO nanoparticles as well as aggregates after the reaction. As can be seen from the images, the nanoparticle size was in good agreement and fell between 80 nm and 180 nm. The mean value of synthesized ZnO NPs was about 124.64 nm from histogram analysis (Fig. 7C). These pictures confirmed the formation of synthesized ZnO nanocrystalline particles.^29,30^ The synthesized particles are roughly spherical in form and cluster into larger particles without a distinct morphology, as these images reveal.

### 3.6. Elemental analysis by EDX

**Fig. 8:**
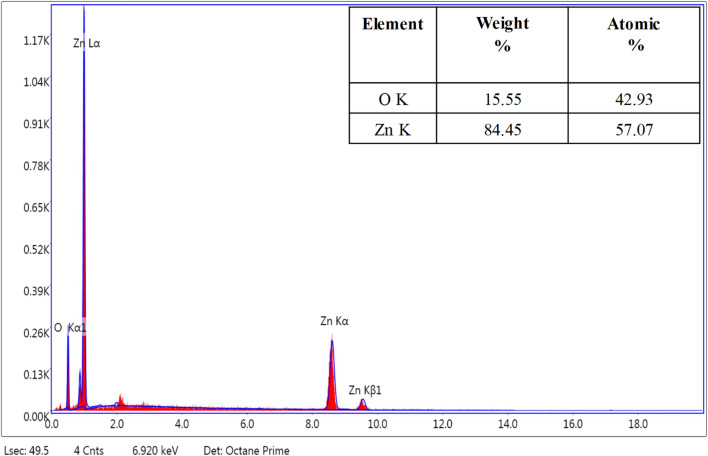
EDX spectra and elemental analysis of the green-synthesized ZnO NPs.

The elemental identification and quantitative compositional information of the synthesized ZnO NPs are displayed by the EDX signal characteristic, in which four peaks were recognized as zinc and oxygen. Strong signals of zinc and oxygen in the examined field were observed. Fig. 8 illustrates that the purity of ZnO powder was good, *i.e.*, Zn content was 84.45%, and oxygen content was 15.55% with very few impurities. Theoretically, expected stoichiometric mass percentages of Zn and O were 80.3% and 19.7% in the previous exploration.^31^ Zinc's composition was higher in the synthesized NPs, which suggested that the synthesized ZnO nanoparticles were nearly stoichiometric. The peaks relate to Zn and O, proving the purity of ZnO nanoparticles obtained through calcination.

### 3.7. Electrochemical analysis

**Fig. 9:**
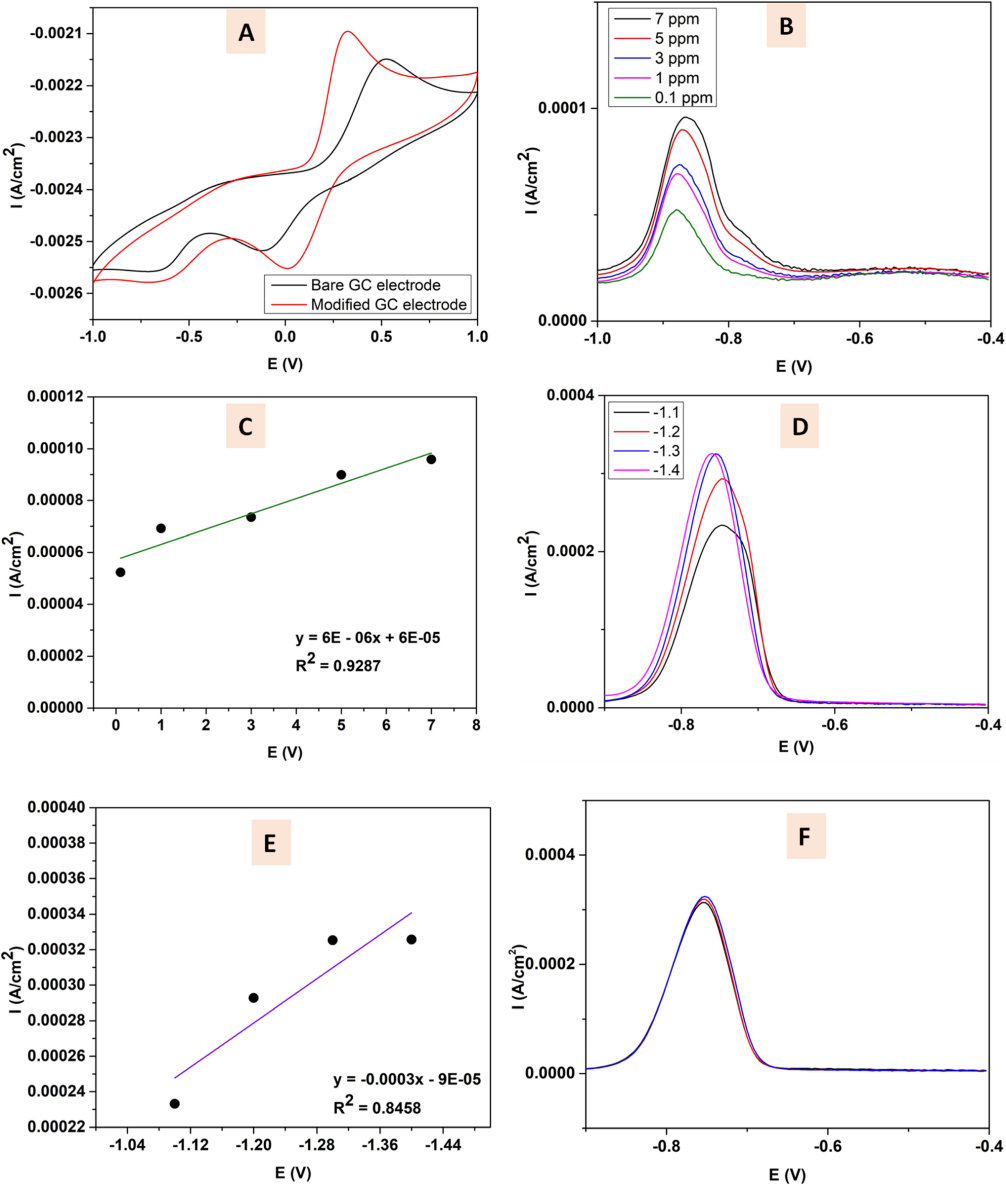
(A) CV of the unmodified GCE and ZnO modified electrode (B) DPASV at deposition potential −1.3 V, condition time 10 s, deposition duration 100 s, and different Cd concentrations in stripping range −1 to −0.4 V, (C) relationship between Cd content and current recorded at stripping range −1 to −0.4 V, condition time 10 s, deposition potential −1.3 V, deposition time 100 s, (D) DPASV for, (E) current-deposition voltage relationship with a given Cd concentration (10 ppm), condition time 10 s, stripping range −1 to −0.4 V, deposition time 100 s, (F) DPASV for three repetition tests with a fixed concentration of Cd concentration (10 ppm), stripping intervals of −1 to −0.4 V, 100 s for deposition, 10 s for condition.

The cyclic voltammetry (CV) of the green-synthesized ZnO NPs modified glassy carbon electrode and bare electrode is depicted in Fig. 9A, which was executed using a mixture of 0.1 M NaCl and 2.5 mM [Fe(CN)_6_]^3−/4−^ with 50 mV s^−1^. The DPASV (Differential Pulse Anodic Stripping Voltammetry) analysis revealed that ZnO (Fig. 9B) showed well-resolved stripping peaks with anodic peak currents (*I*_p,a_) correlating with Cd concentration across the range of 0.1–7 ppm. In that case, a mixture of electrolyte (NaCl) and a heavy metal-containing solution was used for detection purposes. This linear range demonstrated the material's ability to detect both trace and moderate concentrations. The concentration-dependent calibration (Fig. 9C) exhibited a linear relationship with a correlation coefficient (*R*^2^) of 0.93, indicating quite strong linearity, though slightly lower than some advanced MOF-based sensors, suggesting room for optimization through surface modification or nano-structuring approaches. The linear fitting between Cd concentration and *I*_p,a_ showed the regression equation: *I*_p,a_ = 6 × 10^−6^*x* + 6 × 10^−5^ (where *x* is the Cd concentration), yielding a sensitivity of 6 × 10^−6^ A ppm^−1^, which reflects the material's ability to generate measurable current responses per unit concentration change. While this sensitivity is moderate compared to some nanostructured metal–organic frameworks,^32,33^ it remains suitable for practical environmental monitoring applications where cadmium concentrations typically fall within the µg L^−1^ to mg L^−1^ range. The limit of detection (LOD) and limit of quantification (LOQ) were calculated using the 3.3 SD/slope and 10 SD/slope methods, respectively. The LOD was 2.16 ppm, and the LOQ was 6.54 ppm. This detection limit isn't on the same level as some of the state-of-the-art cadmium sensors, but still, these parameters are better compared to some of the recent studies of cadmium sensing using ZnO.^34^ The current density of 9.58 × 10^−5^ A cm^−2^ achieved at 7 ppm cadmium concentration demonstrates substantial electrochemical activity, indicating effective surface utilization and favorable mass transport characteristics. This current density suggests that the ZnO surface provides adequate active sites for cadmium pre-concentration during the deposition step and subsequent oxidative stripping. The analysis time of less than 3 minutes represented a significant advantage for rapid field testing and high-throughput screening applications. This fast response time, combined with the straightforward synthesis of ZnO, makes this material particularly attractive for portable sensor development and on-site environmental monitoring. The deposition potential study (Fig. 9D) was conducted in the range from −1.1 to −1.4 V at a 10 ppm Cd concentration, yielding a peak current of 3.25 × 10^−4^ A cm^−2^ at −1.4 V. The relationship between current response and deposition potential (Fig. 9E) exhibited a linear correlation (*R*^2^ = 0.85), with the regression equation: *I*_p,a_ = −0.0003*x* + 9 × 10^−5^ (where *x* is the Cd concentration). The peak current response at −1.4 V is 1.4 times better compared to the response at −1.1 V. The peak current response for −1.3 V and −1.4 V was almost the same, indicating further increasing the deposition voltage wouldn't necessarily increase sensitivity. The reproducibility studies (Fig. 9F), conducted over three repetitions at 10 ppm Cd^2+^, demonstrated acceptable precision, as the mean peak current shift is minimal. The obtained critical parameters from DPASV are presented in Table 6.

**Table 6:** Calculated parameters from DPASV

Parameter	DPASV
Linear range (ppm)	0.1–7 ppm
Correlation coefficient (*R*^2^)	0.93
Sensitivity (A ppm^−1^)	6 × 10^−6^
LOD (ppm)	2.16
LOQ (ppm)	6.54
*I*_p,a_ at 7 ppm Cd detection (A cm^−2^)	9.58 × 10^−5^
Analysis time (min)	<3

### 3.8. Sensing mechanism

**Fig. 10:**
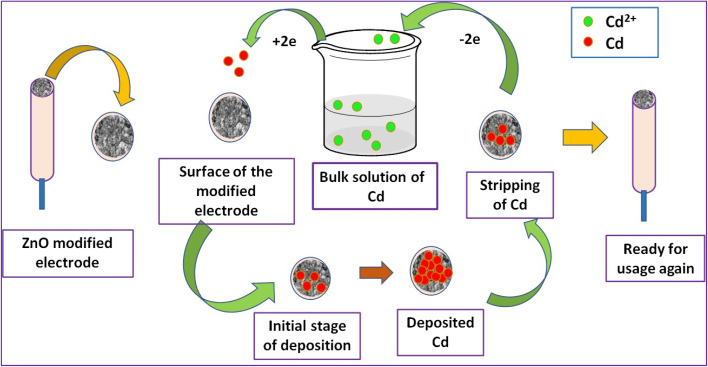
Sensing mechanism of green-synthesized ZnO NPs-modified electrode.

The sensing mechanism mainly consists of deposition and stripping steps. These steps were used to explore Cd detection, employing the DPASV technique. After immersion of the ZnO-modified electrode into the cadmium solution, a layer (Cd^2+^) was formed on the surface of the fabricated electrode through a deposition process. Consequently, Cd^2+^ was reduced to Cd by acquiring electrons.^35,36^Cd^2+^ + 2e^−^ → Cd (s)when stripping began after the deposition process was finished, the deposited metals (Cd) underwent electron release, oxidizing to Cd^2+^ ions. The amount of Cd in the solution was calculated by considering the faradaic current. The overall process is graphically depicted in Fig. 10.Cd (s) → Cd^2+^ + 2e^−^

### 3.9. Discussion

The formation of hexagonal ZnO NPs utilizing bay leaf extract was confirmed by the X-ray diffractogram, where the average crystallite size using several well-known model equations was within the range of 4–42 nm, except the linear straight model of Scherrer's equation (126 nm). In addition, the FTIR spectrum ensured the successful formation of a Zn–O bond in the green-synthesized product, whereas SEM micrographs revealed the smaller spherical particle with a particle size of 124.64 nm (from histogram analysis). As a metal oxide and nanomaterial, ZnO has strong catalytic activity and a high surface area. Generally, nanomaterials with a large surface area are more efficient for electrochemical techniques due to their unique features, such as rapid reaction times, superior activity, and the ability to detect analytes even at lower concentrations.^37^ Moreover, the use of leaf extract for the synthesis of ZnO NPs in place of toxic chemicals enhances the surface area, stability, and conductivity.^38^ Consequently, the green-synthesized material had the capacity to quickly identify heavy metals in an aqueous medium through an electrochemical method in place of conventional methods within a very short amount of time. In this research, the ZnO NPs on GCE acted as an electrochemical sensor, which was able to detect cadmium in aqueous medium with a concentration range of 0.1–7 ppm through the DPASV technique. A comparison study is included in Table 7 between the outcomes obtained from this research and those of previous explorations. So, the bay-leaf-extracted ZnO/GCE sensor developed in this study exhibits a balance of eco-friendly synthesis and analytical performance when benchmarked against existing ZnO-based cadmium sensors. Unlike conventional ZnO composites, *e.g.*, ZnO-PVA-graphene/GCE using SWASV with a broad 0–80 ppm range but high LOD of 9.88 ppm, and ZnO@graphene/SPCE achieving sub-µg L^−1^ detection but lacking green synthesis, our DPASV-based ZnO/GCE offers a moderate linear window (0.1–7 ppm) and a LOD of 2.16 ppm with an LOQ of 6.54 ppm, paired with a sensitivity of 6 × 10^−6^ A ppm^−1^. While some non-green sensors reach ultra-low detection limits-such as ZnO@SiO_2_/SPE with LOD of 4.4 × 10^−11^ mol L^−1^ or Cr-EDTA@ZnO/GCE achieving 1.25 nM - they involve complex fabrication or harmful reagents. By contrast, our synthesized bay-leaf route ensures facile, low-cost, and sustainable production without compromising reproducibility, positioning it as a potential alternative for on-site cadmium monitoring. Moreover, compared to other green approaches like Bi_2_O_3_/CeO_2_/SPE (SWASV, LOD 0.14 µg L^−1^) synthesized *via* serine, the bay-leaf ZnO/GCE sensor reduces processing steps. Consequently, this work not only advances the field of green electrochemical sensing but also provides a robust platform that harmonizes environmental stewardship with analytical efficacy.

**Table 7:** Comparison of electrochemical sensors for cadmium detection^a^

Material/electrode	Detection technique	Linear range (Cd^2+^)	LOD (Cd^2+^)	LOQ (Cd^2+^)	Sensitivity	Green synthesis	Ref.
ZnO/GCE	DPASV	0.1–7 ppm	2.16 ppm	6.54 ppm	6 × 10^−6^ A ppm^−1^	Yes (bay leaf extract)	This work
ZnO-PVA-graphene/GCE	SWASV	0–80 ppm	9.88 ppm	—	—	No	39
ZnO@graphene/SPCE	SWASV	10–200 µg L^−1^	0.6 µg L^−1^	—	—	No	40
ZnO/ErGO/GCE	DPASV	2.5–200 µM	1.69 ppb	—	—	No	41
ZnO/porous-graphene/laser-printed electrode	SWASV	1–25 µM	2.08 nM	—	5.83 µM µA^−1^	No	42
Cr-EDTA@ZnO/GCE	—	0.00125 µM–10 µM	1.25 nM	—	—	No	43
Bi_2_O_3_/CeO_2_/SPE	SWASV	0.5–85 µg L^−1^	0.14 µg L^−1^	—	—	Yes (serine)	44

a Abbreviation: DPASV-Differential Pulse Anodic Stripping Voltammetry and SWASV-Square Wave Anodic Stripping Voltammetry.

## 4. Conclusion

The successful synthesis of ZnO NPs utilizing bay leaf (*Laurus nobilis*) extract is confirmed by several sophisticated instruments, especially from the X-ray diffractogram, which verified that a hexagonal structure with crystallite size from 4 to 42 nm had formed. Small crystal size and smaller spherical particles provide a large surface area, which imparts activity as an electrochemical sensor. As a result, ZnO NPs on GCE performed high sensitivity for Cd detection (0.1–7 ppm) by DPASV. So, to overcome the real-world challenges of minimizing water scarcity, green-synthesized ZnO NPs will be more applicable. But in that case, large-scale production and modification of the properties of ZnO NPs will be the main focus, so that they can detect several heavy metals. Additionally, it can be converted into a portable device using modern techniques, allowing anyone to use it easily. Another benefit is that a small amount of sample is required for electrochemical sensing, making it economically beneficial. So, the research outcome provides a sustainable balance focusing on developing green electrochemical sensors that are not only helpful for the environment but also for living beings through the detection of heavy metals.

## Author contributions

Sumaya Tabassum carried out the manuscript, authored the initial draft, and synthesized the final product. The manuscript was written with assistance from Md. Abu Saeed, Promit Debnath, and Nilufar Yasmin Liza. Sanjida Khan performed the TGA analysis. Muhammad Shahriar Bashar conducted the SEM and EDAX analysis. Suravi Islam checked the manuscript. Samina Ahmed oversaw the project work. Md. Sahadat Hossain performed data analysis and assisted in drafting the manuscript.

## Conflicts of interest

There are no conflicts to declare.

## Data Availability

Data will be made available on request.
